# Use of an electronic administrative database to identify older community dwelling adults at high-risk for hospitalization or emergency department visits: The elders risk assessment index

**DOI:** 10.1186/1472-6963-10-338

**Published:** 2010-12-13

**Authors:** Sarah J Crane, Ericka E Tung, Gregory J Hanson, Stephen Cha, Rajeev Chaudhry, Paul Y Takahashi

**Affiliations:** 1 Division of Primary Care Internal Medicine, Department of Medicine, Mayo Clinic, 200 First Street SW, Rochester, Minnesota, 55905, USA; 2 Division of Biomedical Statistics and Informatics, Department of Health Sciences Research, Mayo Clinic, 200 First Street SW, Rochester, Minnesota, 55905, USA

## Abstract

**Background:**

The prevention of recurrent hospitalizations in the frail elderly requires the implementation of high-intensity interventions such as case management. In order to be practically and financially sustainable, these programs require a method of identifying those patients most at risk for hospitalization, and therefore most likely to benefit from an intervention. The goal of this study is to demonstrate the use of an electronic medical record to create an administrative index which is able to risk-stratify this heterogeneous population.

**Methods:**

We conducted a retrospective cohort study at a single tertiary care facility in Rochester, Minnesota. Patients included all 12,650 community-dwelling adults age 60 and older assigned to a primary care internal medicine provider on January 1, 2005. Patient risk factors over the previous two years, including demographic characteristics, comorbid diseases, and hospitalizations, were evaluated for significance in a logistic regression model. The primary outcome was the total number of emergency room visits and hospitalizations in the subsequent two years. Risk factors were assigned a score based on their regression coefficient estimate and a total risk score created. This score was evaluated for sensitivity and specificity.

**Results:**

The final model had an AUC of 0.678 for the primary outcome. Patients in the highest 10% of the risk group had a relative risk of 9.5 for either hospitalization or emergency room visits, and a relative risk of 13.3 for hospitalization in the subsequent two year period.

**Conclusions:**

It is possible to create a screening tool which identifies an elderly population at high risk for hospital and emergency room admission using clinical and administrative data readily available within an electronic medical record.

## Background

The aging of the United States population represents a demographic imperative for innovation in the provision of healthcare to older Americans. Those aged 65 and older represented 12.4% of the total U.S. population in 2005, but this number is projected to double in the next twenty-five years [1]. Accordingly, the population of older adults at high risk for hospitalization, nursing home placement or functional decline is also increasing, creating an enormous financial and capacity burden on the health care system.

Multiple interventions, such as case management and transition management programs, have target the prevention of recurrent hospitalizations among community dwelling older adults, and are under great scrutiny in the arenas of research and policy development [2-5]. The complexity and cost of many of these interventions, combined with the demographic challenges, require that the investment of these resources be made in the patient population that is most likely to benefit. In order to identify those patients, health care providers require some form of risk assessment to focus their efforts - recognizing that the elderly population is very heterogeneous in function and disease burden.

These challenges have led to the need for a predictive instrument that is accurate, easy to calculate, inexpensive, and does not require patient completion. Our group hypothesized that we could identify older adults at high risk for hospitalization or emergency department visits using only information readily available from a centralized electronic health record, without taking time away from staff and patients. This model is becoming increasingly feasible as national policy continues to strongly encourage the creation and use of electronic medical records. Hospitalization and emergency department encounters were chosen as independent outcomes, as both events are associated with premature institutionalization and high resource utilization [6,7]. The primary aim of this study was to demonstrate that readily accessible information available in a provider's electronic medical record could be used to identify a population of community dwelling older adults at high-risk for hospitalization or emergency room utilization.

## Methods

The study was a retrospective cohort of all patients age 60 and greater who were impaneled on January 1, 2005, in the Division of Primary Care Internal Medicine (PCIM) at Mayo Clinic in Rochester, MN. This division of the Department of Medicine serves local residents, Mayo Clinic employees, and their dependents. Rochester is a city of approximately 100,000 and is surrounded by small rural communities. There are only two other major alternative primary care providers for older adults in this community; the Department of Family Medicine at Mayo Clinic and the Olmsted Medical Group.

### Study Subjects

All adults age 60 and older, assigned to a PCIM primary care provider on January 1, 2005, were included in the analysis. All subjects were community dwelling or lived in an assisted living facility within Olmsted County, MN.

Patients who were residing within a skilled nursing facility on January 1, 2005, were excluded from the study. Patients who did not give consent for their medical chart review were also excluded from analysis, in accordance with Minnesota state law.

### Data Collection

Information was electronically abstracted from the electronic medical record and administrative databases within Mayo Clinic's health records system. Mayo Clinic maintains all electronic medical record information within one system, including hospital, emergency room, nursing home, and clinic-visit information. No individual chart abstraction was performed.

The demographic predictor variables collected included: date of birth, gender, marital status, race, and the number of hospital admission days in the prior two years (January 1, 2003 to December 31, 2004). Hospital days were stratified into two risk groups: one to five and six or more. Age was stratified into categories of 60 to 69, 70 to 79, 80 to 89, and greater than 90.

Comorbid medical illnesses included the presence or history of diabetes mellitus, coronary artery disease (CAD), congestive heart failure (CHF), stroke, chronic obstructive pulmonary disease (COPD), history of cancer, history of hip fracture, and dementia. History of cancer excluded non-melanomatous skin cancers. Diagnoses were identified using ICD-9 billing codes entered by physicians during both inpatient and outpatient encounters. These comorbidities were chosen via consensus discussion based on their known risk for recurrent hospitalizations and greater complexity of care.

The primary outcome variable was the total number of hospitalizations or emergency room visits measured from the date of January 1, 2005, through December 31, 2006. Emergency room visits resulting in a direct hospital admission were recorded as a single outcome event. The total number of hospital admissions and admission days during the same two-year period were collected as secondary outcome measures.

### Data Analysis

Predictor variables for the primary outcome of the total number of hospitalizations or emergency room visits were screened for further analysis using univariate regression models and 1-way ANOVA. The variables with a p-value greater than 0.05 were discarded. A final multivariable regression model using stepwise elimination was then constructed with only those significant predictors identified by the univariate stage. The category of "unknown" race was a significant univariate predictor, but was not included in the final model as the category was not large enough (5%) to statistically influence the final multivariable model and it proved difficult to act upon prospectively in identifying new, at-risk patients.

A total risk score for each individual was calculated based on the significant risk factors using regression estimates multiplied by ten in order to generate manageable scores. The scores were divided by quartiles and the top quartile further divided into the top 10% and then the next 15% (75% to 90%). This split was chosen in an attempt to create categories in the highest risk groups with small enough populations to enable focused future interventions.

To estimate the precision of the score assignment, bootstrapping was used to draw 450 random samples from the original 12,650 patients with replacement. This method provides robust estimates of the standard error of a population parameter such as a regression coefficient[8]. For every sample, a regression model was run using the same predictive variables. The estimate of each predictor in the validation model was the mean of the regression coefficients of each predictor from 450 runs. The standard error was obtained from the standard error of the mean estimates.

1-way ANOVA for mean, Wilcoxon rank sum tests for median and Pearson chi-square test for frequency were used to compare variables across the 5 score categories. Hospitalizations and emergency visits within 2 years were compared across score categories using logistic regression analysis to provide odds ratios. Receiver operating characteristic (ROC) curves were developed to show sensitivity and specificity of hospitalization or emergency visits in 2 years stratified by the risk score.

All information was directly entered via electronic abstraction into a Microsoft Excel (version 2003, Microsoft, Redmond, WA) spreadsheet for data entry, data retrieval, and analysis. The investigators analyzed the final information using SAS 9.1 (Cary, NC).

The Mayo Clinic Institutional Review Board (IRB) reviewed and approved the protocol. All aspects of the research on this project were made in accordance with the principles of the Declaration of Helsinki. The investigators also adhered to Minnesota state statues regarding medical record use and privacy.

## Results

The only variables excluded by the univariate estimates were gender and history of hip fracture, and race, which were not found to be statistically significant. The final estimates from the multivariable model are described in Table 1, along with their associated scores. The estimate, standard deviation and score for the validation model are also presented in Table 1.

**Table 1:** Regression Estimates and Scoring of Predictive Risk Factors: Original Model and Bootstrapping Validation Model

	Original Model				**Validation Model**^**1**^		
	**Regression Estimate**	**Standard Error**	**P-value**	**Score**	**Regression Estimate**	**Standard Error**	**Score**

Married	-0.12	0.03	<. 01	-1	-0.12	0.04	-1

Age 70-79	0.11	0.04	<. 01	1	0.11	0.03	1

Age 80-89	0.31	0.04	<. 01	3	0.31	0.05	3

Age 90 or more	0.67	0.08	<. 01	7	0.67	0.10	7

1-5 hosp days in 2003 or 2004	0.55	0.04	<. 01	5	0.54	0.05	5

6 or more hosp days in 2003 or 2004	1.10	0.05	<. 01	11	1.10	0.07	11

History of Diabetes	0.17	0.04	<. 01	2	0.17	0.04	2

History of CAD/MI/CHF	0.31	0.04	<. 01	3	0.31	0.04	3

History of Stroke	0.23	0.05	<. 01	2	0.23	0.06	2

History of COPD	0.47	0.05	<. 01	5	0.48	0.07	5

History of Cancer	0.10	0.04	<. 01	1	0.10	0.04	1

History of Dementia	0.31	0.05	<. 01	3	0.31	0.06	3

^1^Estimate of each predictor is the mean of the regression coefficients of each predictor from 450 samples. Reported standard error is the standard error of the mean estimates.

There were a total of 13,457 patients in the age range 60 and over in the PCIM panel on January 1, 2005. Ninety-four percent of patients provided consent for medical record review for a total study population of 12,650 patients. The scores based on the instrument ranged from -7 to 32. The patients were placed in five groups based on total score, with the lowest quartile scores ranging from -7 to -1, the 2nd quartile 0 to 3, 3rd quartile 4 to 8, 75% to 90% group 9 to 15, and the top 10% had scores of 16 and greater. The average age in the top 10% by score was 80.7 years, compared to 65.0 years in the bottom quartile (P < 0.001). All comorbid conditions had significantly higher proportions in the highest 10%, compared to the lowest quartile as described in Table 2.

**Table 2:** Characteristics of the Population by Quartile and top 10%

Variable	-7:-1	0:3	4:8	9:15	16+	P-
		
	N = 2106	N = 4114	N = 3115	N = 2129	N = 1186	value
Age (± SD)	65.0(4.3)	70.9 (6.9)	74.2 (8.4)	77.4 (9.3)	80.7 (8.4)	<. 01

Age, n (%)						<. 01

• Age 60-69	1930 (92)	1778 (43)	1014 (33)	470 (22)	135 (11)	

• Age 70-79	148 (7)	1882 (46)	1186 (38)	740 (35)	327 (28)	

• Age 80-89	28 (1)	446 (11)	842 (27)	681 (32)	531 (45)	

• Age >90	0 (0)	8 (0)	73 (2)	238 (11)	193 (16)	

Female, n (%)	1193 (57)	2500 (61)	1732 (56)	1159 (54)	683 (58)	<. 01

Stayed in a hospital (2003-2004), n (%)	18 (1)	1 (0)	924 (30)	1437 (67)	1160 (98)	<. 01

Total hospital days (2003-2004), Median (Min, Max)	0 (0, 4)	0 (0, 5)	0 (0, 49)	2 (0, 123)	9.5 (0, 153)	<. 01

Lived in a NH (2003-2004), n (%)	14 (1)	133 (3)	290 (9)	420 (20)	496 (42)	<. 01

Previous history ever of NH stay, n (%)	27 (1)	323 (8)	536 (17)	669 (31)	706 (60)	<. 01

History of Diabetes, n (%)	104 (5)	794 (19)	964 (31)	726 (34)	494 (42)	<. 01

History of CAD/MI/CHF, n (%)	50(2)	533 (13)	1268 (41)	1207 (57)	913 (77)	<. 01

History of Stroke, n (%)	20 (1)	154 (4)	421 (14)	480 (23)	472 (40)	<. 01

History of COPD, n (%)	14 (1)	19 (0)	408 (13)	553 (26)	484 (41)	<. 01

History of Cancer, n (%)	76 (4)	927 (23)	851 (27)	686 (32)	448 (38)	<. 01

History of Hip Fracture, n (%)	11 (1)	59 (1)	98 (3)	120 (6)	144 (12)	<. 01

History of Dementia, n (%)	17 (1)	139 (3)	396 (13)	434 (20)	366 (31)	<. 01

Marital Status, n (%)						

• Married	1961(93)	2719 (66)	1920 (62)	1142 (54)	521 (44)	

The primary outcome was the number of emergency room and hospital visits in the subsequent two years, January 1, 2005, to December 31, 2006. The number of total visits/admissions increased consistently with an increasing risk score as described in Table 3. This was significant with a P-value < 0.01. The relative risk of the primary outcome of total ER visits and hospital stays also increased significantly between each of the risk categories.

**Table 3:** Total Number and Relative Risk of Total Emergency Room Visit and Hospital Stay, Emergency Room Visit Alone and Hospital Stay Alone by Risk Category in Two Years Follow-Up (2005-2006)

Score	Total ER/Hospitalizations N (%)	Mean (SD)	Median (Range)	Relative Risk of ER Visit or Hospital Stay OR (95% CI)	Relative Risk of ER Visit OR (95% CI)	Relative Risk of Hospital Visit OR (95%CI)
**-7:-1**	518 (25)	0.4 (0.8)	0 (0,8)	0.0 (Reference)	0.0 (Reference)	0.0 (Reference)

**0:3**	1549 (38)	0.7 (1.1)	0 (0,13)	1.85 (1.6-2.1)	1.7 (1.5-2.0)	1.9 (1.6-2.2)

**4:8**	1507 (48)	1.1 (1.6)	0 (0,14)	2.9 (2.5-3.2)	2.3 (2.0-2.6)	3.4 (2.9-3.9)

**9:15**	1314 (62)	1.6 (2.2)	1 (0,24)	4.9 (4.3-5.6)	3.4 (3.0-4.0)	6.0 (5.1-7.0)

**16 +**	897 (76)	2.6 (2.9)	2 (0,24)	9.5 (8.1-11.2)	4.6 (3.9-5.4)	13.3 (11.2-15.9)

**P value**	<. 01	<. 01	<. 01			

**Figure 1:**
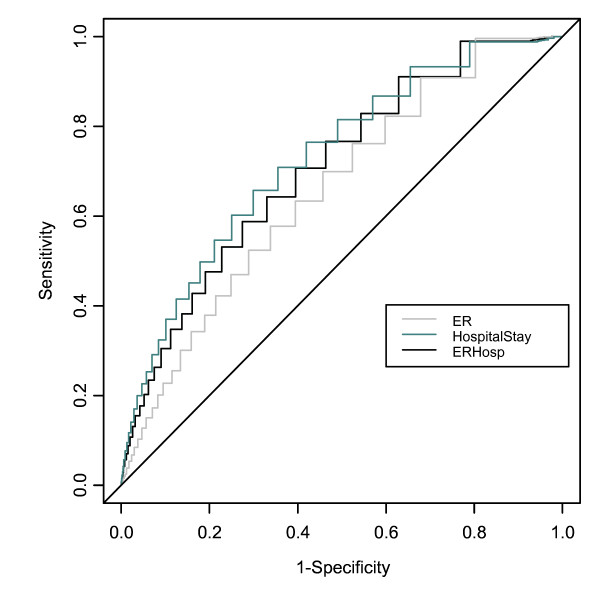
ROC Curves for Total Emergency Room Visit and Hospital Stay, Emergency Room Visit Alone and Hospital Stay Alone

The receiver operating characteristic (ROC) curves associated with the main combined outcome, and the ER and hospital visits individually are described in Figure 1. The area under the curve (AUC) for the primary outcome of combined hospitalizations and emergency room visits was 0.678. For hospital visits only, the AUC was 0.705. For emergency room visits only, the AUC was 0.640.

The results of secondary outcomes evaluated using the risk score included two-year (2005 and 2006) total number of hospital admissions and number of days hospitalized. Each of these outcomes increased significantly with increasing risk score as described in Table 4.

**Table 4:** Total Number of ER Visits/Hospital Admissions and Hospital Days By Risk Category in Two Years Follow-Up (2005-2006)

Score	# of admissions (2 yrs)		# of hospital days (2 yrs)	
	
	Mean/SD	Median/Range	Mean/SD	Median/Range
**-7:-1**	0.4 ± 0.8	0(0, 8)	0.6 ± 3.5	0(0, 102)

**0:3**	0.7 ± 1.2	0(0, 13)	1.4 ± 5.2	0(0, 152)

**4:8**	1.1 ± 1.6	0(0, 14)	2.4 ± 6.3	0(0, 134)

**9:15**	1.6 ± 2.2	1(0, 24)	4.1 ± 9.0	0(0, 132)

**16 +**	2.6 ± 2.9	2(0, 24)	8.0 ± 13.3	4(0, 142)

**P-value**	<. 01	<. 01	<. 01	<. 01

## Discussion

In this study, a prognostic index was developed and validated, based on a scoring system that derived information from community-dwelling elderly patients' electronic medical records. The Elders Risk Assessment (ERA) index accurately identified older adults at high-risk of emergency department encounters and hospitalization; two outcomes that can lead to significant morbidity, functional decline, and institutionalization [7].

Previous authors have developed screening instruments aimed at identifying high risk populations of older adults. The ERA was developed to address and overcome a number of barriers that are typically associated with these instruments.

One of the primary barriers is the requirement for patient self-reporting of information. The best validated self-administered prognostic index is the Probability of Repeated Admissions (PRA) [9,10]. This eight-item tool has been widely used by managed care organizations to prospectively identify enrollees at risk for repeated hospital admissions and health care resource utilization. This instrument has been shown to have good discriminating ability for one-year risk of hospitalization, with reported areas under the ROC curves ranging from 0.620-0.696, depending on the validation population and setting [11-13]. Similarly, the Community Assessment Risk Screen (CARS) index identifies those older adults at increased risk of hospitalization or emergency department visits with self-reported information about medical conditions, medication use, and health service utilization. Utilizing this risk classification, Shelton and colleagues found that the area under the ROC curve to be 0.74 for hospitalization or emergency department visits [6]. Mazzaglia and colleagues utilized self-reported data (functional status, sensory impairment, unintentional weight loss, and use of home care services), from community dwelling older adults in Florence, Italy, to create a risk score that was also found to be predictive of hospitalization (in the subsequent 15 years) with AUC of 0.68 [14]. Unfortunately, low response rates [13], recall bias [15], literacy requirements [16], time, and cost [17] have proven to be significant barriers to widespread use of self-reported instruments. Response rates for the PRA have ranged from 50-60% in the managed care setting [13,17]. A major advantage of the ERA index is that it uses administrative data, which is unaffected by the aforementioned limitations which are intrinsic to self-reported data.

The ERA also performed favorably when compared with the administrative or "proxy" PRA. The administrative PRA model derives information from a health plan's multiple databases including a pharmacy database, chronic disease registries, billing data, and utilization data registries to calculate a risk score which performs similarly to the original self-reported Pra (AUC 0.694 vs. 0.696) in predicting hospitalization [11]. While undoubtedly useful in the managed care setting, this proxy model is challenging to adopt in traditional fee-for-service medical practices, like ours, which serves patients who utilize a multitude of pharmacies and supplemental insurance carriers thus limiting access to those database sources.

Combined hospitalization and emergency room visits were chosen as the primary outcome because they are early precursors to the functional decline and institutionalization, which it is our goal to prevent. They also often result from acute changes in chronic conditions such as COPD, where early intervention by an outpatient provider may prevent recurrent admissions. In an effort to improve the primary care physician's awareness of these risks, we have subsequently developed it for real time use among our primary care providers in our electronic environment with a software system called Generic Disease Management Systems (GDMS). GDMS is a web-based application developed by Mayo Clinic and the Netherlands-based Noaber Foundation, which uses GE Web Services and a MSQweb.net platform to retrieve patient vital statistics such as blood pressure, weight, body mass index, age, demographic information, prior diagnoses, allergies, prior radiology diagnostic tests, and previous preventive services (eg, immunizations, cancer and metabolic screenings, laboratory test results pertaining to diabetes, coronary artery disease, asthma, and depression) from different clinical information systems. The ERA score is now calculated in real time based on the scoring system described in this article and displayed on the GDMS print out that we include in the rooming packet for all our patient visits. This allows our providers to easily identify at-risk elders and to pay special attention to the patient if clinically needed.

This ability to measure ERA scores in real time is now being further developed into a registry which allows us to identify these high-risk patients as a unique population, similar to the population-based systems used to manage diabetics. Currently, this real-time registry is allowing the implementation and measurement of interventions such as transitions programs, discussions regarding goals of care, appointment access prioritization, and accelerated triage aimed at preventing recurrent admissions and secondary functional decline.

This study is not without methodological limitations. First, the patient information obtained from administrative databases was recorded prior to the outcome of interest for purposes other than investigation of our hypothesis. Coding data were utilized to identify whether individuals had been diagnosed with any of the six predictor comorbid conditions. Coding data may under-estimate secondary diagnoses, however, other authors have found that administrative data such as ICD-9 codes, typically correlate well with patient chart diagnoses [18].

Second, this study was a retrospective cohort analysis. This creates the possibility of underreported risk factors, as well as outcomes. Although most patients receive both their acute and chronic care from Mayo Clinic, as their primary provider, it is certainly possible that they could have hospitalizations or chronic diagnoses which are identified elsewhere and of which our electronic medical record is therefore unaware. Although the outcome data requires further prospective validation, the retrospective collection of risk factor variables is an essential component of the model design and one of the factors this hypothesis was designed to examine.

Third, we did not include functional-status measures in our initial predictive modeling. Functional-status measures are known to be independently associated with hospitalization and emergency department visits, however, functional-status data is dependent on patient-provided history or clinician-administered performance testing and is neither routinely collected, nor easily extractible from administrative data [19-21]. Additionally, self-reported information such as functional status and medications, fluctuate throughout an individual's life, further challenging the accurate collection and maintenance of this data. Despite the fact that the functional status was not utilized in our final model, the ERA index compared favorably with the aforementioned indices in which it was included.

## Conclusions

Despite these limitations, results from this study suggest that the ERA index represents a risk identification model, which is an example of an effective, inexpensive, electronic mechanism able to identify populations of older, community-dwelling adults who are at increased risk for hospitalization and emergency department encounters. Administrative and clinical data modeling may afford busy primary care practices or payor organizations the opportunity to identify high-risk populations so that they may effectively allocate resources and evidence-based preventive interventions to those individuals with the greatest need and greatest potential to benefit.

## Competing interests

The authors declare that they have no competing interests.

## Authors' contributions

SJC participated in study design and drafted the manuscript. PT participated in study design and assisted in drafting the manuscript. GH participated in study design and assisted in drafting the manuscript. ET participated in study design and assisted in drafting the manuscript. SC performed the statistical analysis. RC created the GDMS software program for implementing the model and assisted in drafting the manuscript. All authors reviewed and approved the final manuscript.

## Pre-publication history

The pre-publication history for this paper can be accessed here:

http://www.biomedcentral.com/1472-6963/10/338/prepub
